# Adipose tissue from subjects with type 2 diabetes exhibits impaired capillary formation in response to GROα: involvement of MMPs-2 and -9

**DOI:** 10.1080/21623945.2022.2070949

**Published:** 2022-05-12

**Authors:** Yifat Amir Levy, Theodore P Ciaraldi, Sunder R. Mudaliar, Susan A. Phillips, Robert R. Henry

**Affiliations:** a Center for Metabolic Research, Veterans Affairs San Diego Healthcare System, San Diego, CA, USA; b Departments of Medicine, University of California, La Jolla, CA, USA; c Departments of Pediatrics, University of California, La Jolla, CA, USA

**Keywords:** Type 2 diabetes, angiogenesis, adipose tissue, myokines, MMPs, groα

## Abstract

Type 2 Diabetes (T2D) is associated with impaired vascularization of adipose tissue (AT) . IL8, GROα and IL15 are pro-angiogenic myokines, secreted at elevated levels by T2D myotubes. We explored the direct impact of these myokines on AT vascularization. AT explants from subjects with T2D and without diabetes (non-diabetic, ND) were treated with rIL8, rGROα and rIL15 in concentrations equal to those in conditioned media (CM) from T2D and ND myotubes, and sprout formation evaluated. Endothelial cells (EC) were isolated from T2D and ND-AT, treated with rGROα and tube formation evaluated. Finally, we investigated the involvement of MMP-2 and −9 in vascularization. ND and T2D concentrations of IL8 or IL15   caused similar stimulation of sprout formation in ND- and T2D-AT. GROα exerted a similar effect in ND-AT. When T2D-AT explants were exposed to GROα, sprout formation in response to T2D concentrations was reduced compared to ND. Exposure of EC from T2D-AT to GROα at T2D concentrations resulted in reduced tube formation. Reduced responses to GROα in T2D-AT and EC were also seen for secretion of MMP-2 and −9. The data indicate that skeletal muscle can potentially regulate AT vascularization, with T2D-AT having impairments in sensitivity to GROα, while responding normally to IL8 and IL15.

## Introduction

Obesity, pre-diabetes, and Type 2 Diabetes (T2D) lie along a continuum of metabolic disease. A common feature is insulin resistance in peripheral tissues, primarily skeletal muscle, adipose tissue, and liver [1].

Capillaries play a crucial role mediating the supply of oxygen, glucose and other substrates, hormones, and inflammatory cells, from the circulation to adipose tissue (AT) [2]. Inappropriate capillarization of AT may limit the delivery of the above factors, lead to relative hypoxia and a subsequent inflammatory response that exacerbates metabolic dysfunction [3,4]. The importance of altered vascularization in insulin-resistant states is supported by the observations that capillary density (CD) is reduced in AT from obese individuals [3,5]. Two major factors determine the vascular phenotypes of AT. One is the intrinsic character of local endothelial cells (EC) [6,7], and another is the microenvironment of pro- and anti-angiogenic factors within each tissue [4,8,9].

Over the past two decades, evidence has accumulated that skeletal muscle (SkM) is a secretory tissue that releases a variety of myokines which can act in autocrine, paracrine and/or endocrine fashions to modulate multiple processes in other tissues (reviewed in [10]). While some of the secreted factors are unique to SkM, many are broadly produced and secreted cyto- and chemokines and growth factors. Among them are IL8, GROα and IL15, which while acting as modulators of inflammation (11), are known also to regulate angiogenesis [11–13]. Our laboratory and others have reported that myotubes from human subjects with T2D secrete higher levels of IL8, GRO and IL15 compared to ND myotubes [14,15]. In addition, we reported that the elevated levels of IL8 secreted from T2D myotubes impaired angiogenesis in both human umbilical vein endothelial cells (HUVEC) and human skeletal muscle explants when compared to levels from ND myotubes [16].

In the current report, we investigate the potential effects of the elevated levels of IL8, GROα and IL15 secreted from T2D SkM on capillarization of AT from ND and T2D subjects. As a surrogate for angiogenesis in AT, we employed adipose tissue explants, which can form endothelial sprouts, and EC isolated from AT. Understanding the roles of IL8, GROα, and IL15 in regulating angiogenesis and the signalling pathways involved could shed light on their ability to modulate vascularization and the metabolic function of AT in T2D.

## Results

### Effects of exogenous myokines on capillary length in adipose tissue explants

**Figure 1.**
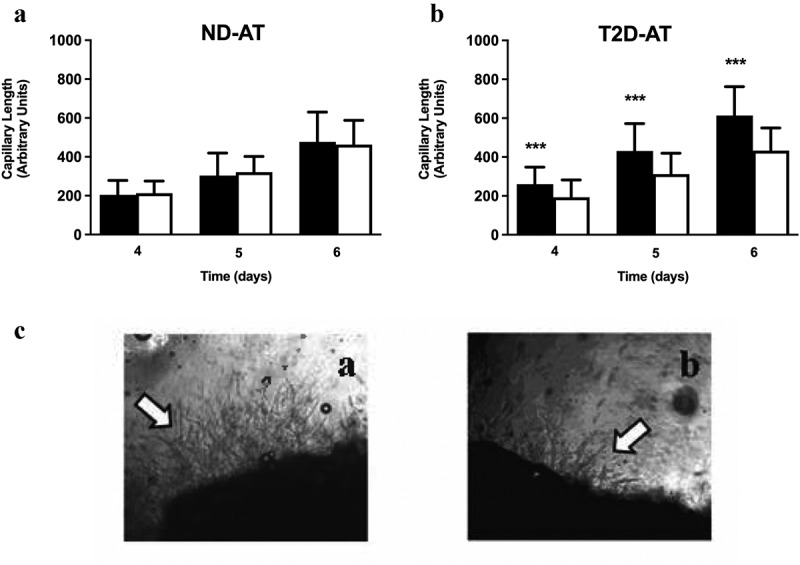
Effects of ND- and T2D [GROα] on capillary outgrowth from ND- and T2D-AT.

To explore whether higher levels of pro-angiogenic factors IL8, IL15 and GROα could be involved in altered capillary sprout formation in AT, rIL8, rGROα and rIL15, either alone or in combination, were added to ND-AT and T2D-AT explants in concentrations equal to the averages of those found in CM from T2D and ND myotubes ([T2D-IL8] = 3280 and [ND-IL8 = 2071 pg/mL; [GROα] = 4620 and 1859 pg/mL; and [IL15] = 1.07 and 0.6 pg/mL, respectively) [14]. Capillary outgrowth was measured after 4–6 days. Capillary outgrowth was stimulated to a similar extent in ND-AT and in T2D-AT by the rIL8 + rGROα + rIL15 combination at ND and T2D concentrations (Supplementary Material, Figure 1a, b). The same was true after exposure to rIL15 alone; no difference between groups or concentrations (SM, Figure 1c, d). Regarding rIL8, after 5 days of treatment, capillary outgrowth in ND-AT was modestly, but significantly (p < 0.05), lower in response to the T2D concentration of IL8 compared to ND-IL8. This difference was lost by day 6 of treatment. In T2D-AT, the response was the same to both concentrations of IL8 (SM, Figure 1e, f).

A & B. Quantification of capillary length from AT explants from ND (A) or T2D (B) subjects after incubation with rGROα in concentrations equivalent to ND- (solid bars) or T2D (open bars) myotube CM for 4d – 6d. Average + SD, ND-AT, n = 14 subjects and T2D-AT n = 15. Shown is the average capillary length/500μm^2^ for three fields of at least three different explants/subject. ***p < 0.005 T2D-rGROα vs. ND- rGROα. C. Representative images of adipose tissue explants from T2D-AT after incubation with rGROα in concentrations equivalent to ND- (a) or T2D-CM (b).

Finally, when exposed to rGROα alone, ND-AT responded the same to both levels of rGROα (Figure 1a). However, T2D-AT displayed a differential sensitivity to rGROα, with treatment by higher (T2D) concentrations suppressing capillary outgrowth compared to treatment at the lower ND-CM level (Figure 1b) (p < 0.005 at each day 4–6). Figure 1c presents representative images of capillary outgrowth from T2D-AT explants after exposure to ND- (Figure 1 c-a) and T2D-CM (Figure 1 c-b) concentrations of rGROα. These results suggest that higher levels of GROα such as those secreted from T2D myotubes might contribute to the lower capillarization observed in T2D-AT [5].

### Effects of exogenous GROα on tube formation by endothelial cells isolated from ND- and T2D-AT

**Figure 2.**
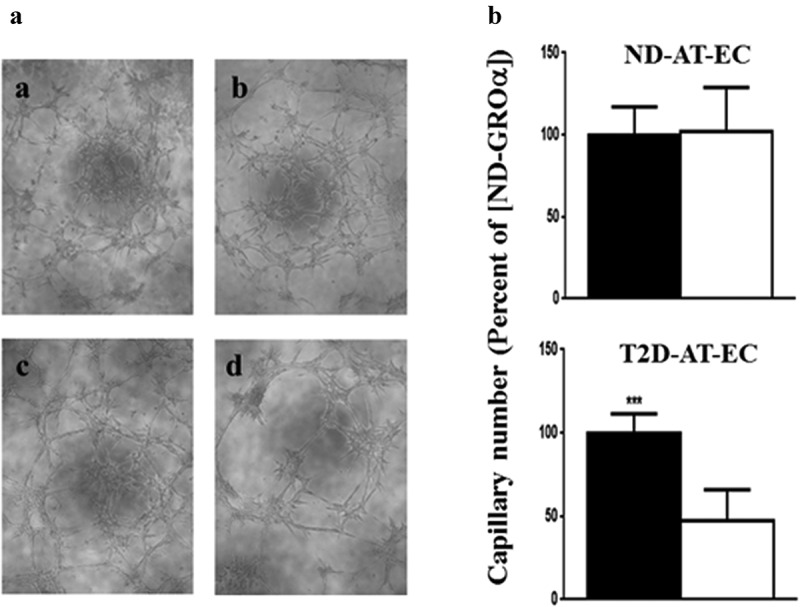
Effects of ND- and T2D-[rGROα] on capillary tube formation by endothelial cells isolated from ND- and T2D-AT. A. Representative images of tube formation of endothelial cells isolated from adipose tissue explants from ND-AT (a, b) or T2D-AT (c, d) after incubation with rGROα in concentrations equivalent to ND-CM (a, c) or T2D-CM [rGROα] (b, d).

Adipose tissue explants represent a mixed system, where multiple cell types may contribute pro- and anti-angiogenic factors to the microenvironment in addition to those provided by myotubes. To understand more about the direct effects of myotube-produced levels of GROα on AT angiogenesis, endothelial cells were isolated from AT of ND (ND-AT-EC) and T2D (T2D-AT-EC) subjects. These AT-EC display characteristic endothelial markers Von Willebrand Factor and CD31 (SM, Figure 2b & 2d) and can form tube-like structures when stimulated, like established endothelial cell lines (SM, Figure 2e) [17].

B. Quantification of tube formation of endothelial cells isolated from adipose tissue explants from ND (top) or T2D (bottom) subjects after incubation with rGROα in concentrations equivalent to ND- rGROα (solid bars) or T2D- rGROα (open bars). Average + SD, ND-AT-EC, n = 4 subjects and T2D-AT-EC n = 6. Shown is the average capillary number/500μm^2^ for 4 fields of at least two independent experiments for each subject. ***p < 0.005 T2D-rGROα vs. ND-rGROα.

Like the procedure with AT explants, AT-EC were exposed to concentrations of rGROα equal to those present in media conditioned by ND- and T2D-CM myotubes. Since the readout in this assay is the formation of tube-like structures by existing cells, rather than capillary outgrowth, treatment times could be shortened (to 8 hr rather than days). When treated with rGROα, the response of ND-AT-EC was very similar to that of ND-AT, with equal stimulation of the angiogenic response at both concentrations (Fig. 2Aa & b and B top panel). However, T2D-AT-EC behaved in the same manner as T2D-AT, where the higher concentration of GROα suppressed tube formation, compared to the ND level (Figure 2 a c) & (d and b bottom panel) (p < 0.005).

### MMP-2 & MMP-9 and GROα in regulation of angiogenesis in T2D-AT and T2D-AT-EC

**Figure 3.**
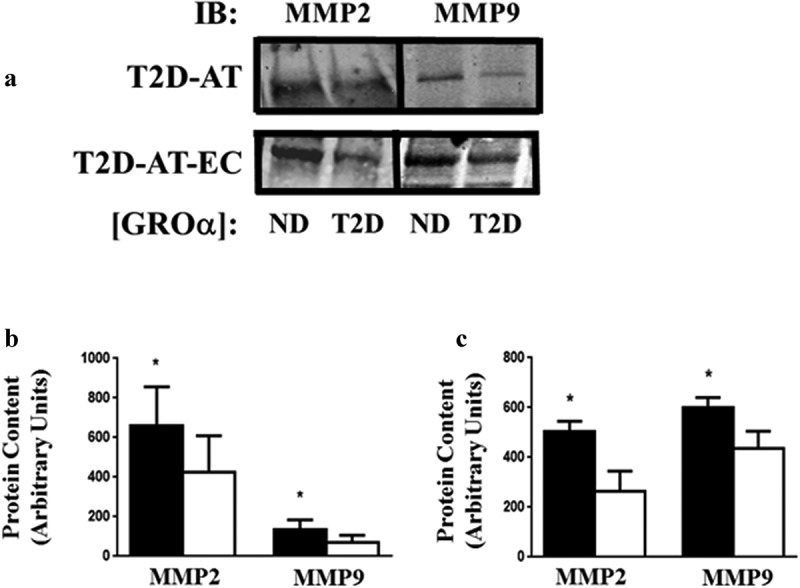
Effects of rGROα on secretion of MMP-2 and MMP-9 from T2D-AT explants and endothelial cells isolated from T2D-AT rGROα in concentrations equivalent to ND- or T2D- GROα was added to T2D-AT and T2D-AT-EC. Culture media was collected from T2D-AT after 4d and from T2D-AT-EC after 8 h. A. Representative western-blots of MMP-2 and MMP-9 from T2D-AT and T2D-AT-EC. B & C. Quantification of blots for MMP-9 and MMP-2 content in the media of T2D-AT (b) and T2D-AT-EC (c); ND-rGROα (solid bars) and T2D-rGROα (open bars). Results are the average + SD of 2 independent experiments for each subject: T2D-AT n = 6, and T2D-AT-EC n = 6. *p < 0.05, T2D-rGROα vs. ND-rGROα.

A critical step in the angiogenic process is the release of matrix metalloproteinases (MMPs) from endothelial cells, with MMP-2 and −9 playing important roles in supporting and promoting angiogenesis [18]. To identify possible molecular mechanisms for the dose-dependency of the T2D-AT angiogenic response to GROα, T2D-AT explants were treated with [ND-GROα] and [T2D-GROα] for 4d and the levels of MMP-2 and MMP-9 released into the media measured. Exposure to the higher concentration of GROα, [T2D- GROα], resulted in significantly less MMP-2 and MMP-9 in the media (Figure 3 a and b) compared to the response to [ND-GROα] (p < 0.01).

Similar responses were detected when T2D-AT-EC were treated with [ND-GROα] and [T2D-GROα] for 8 h and the levels of MMP-2 and MMP-9 released into the media measured. Exposure to the higher concentration of GROα ([T2D- GROα]) resulted in significantly less MMP-2 and MMP-9 released into the media (Figure 3 a and c) compared to the response to [ND-GROα] (p < 0.01).

**Figure 4.**
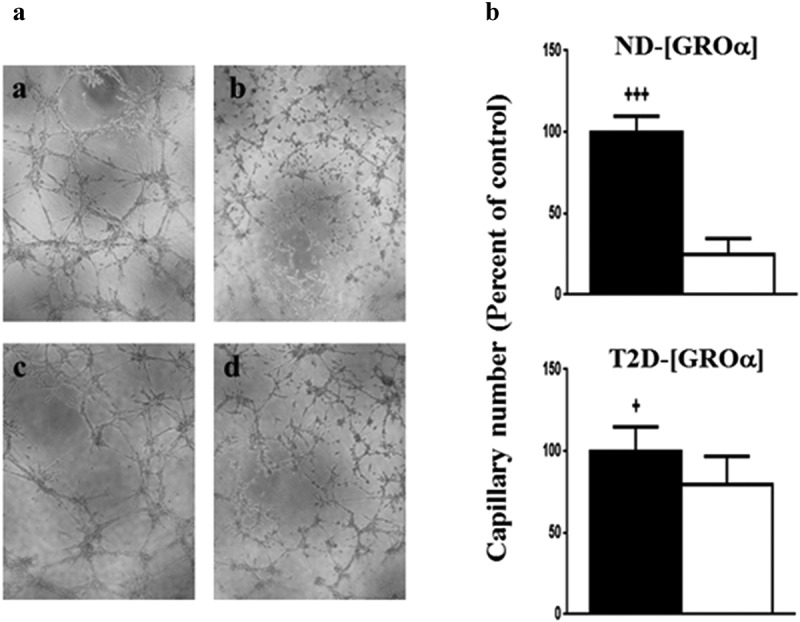
Effects of MMP-2/MMP-9 inhibition with BiPS on capillary tube formation of T2D-AT-EC induced by rGROα. A. Representative images of tube formation of T2D-AT-EC after incubation with rGROα in concentrations equivalent to ND- rGROα (a, b) or T2D- rGROα (c, d), with 0 (a, c) or 25 μM BiPS (b, d), for 8 h. B. Quantification of tube formation of T2D-AT-EC after incubation with rGROα in concentrations equivalent to ND- rGROα (top) or T2D- rGROα (bottom) with 0 (solid bars) or 25 μM BiPS (open bars). Average + SD, T2D-AT-EC n = 3 subjects. Shown is the average capillary number/500μm^2^ for 3 fields of at least 2 independent experiments for each subject. +++p < 0.005 ND-rGROα with 25 μM BiPS vs. ND-rGROα without BiPS, +p < 0.05 T2D-rGROα with 25 μM BiPS vs. T2D-rGROα without BiPS.

Addition of the MMP-2/MMP-9 inhibitor BiPS (25 μM) reduced tube formation in response to both [ND- GROα] (p < 0.0001) and [T2D- GROα] (p < 0.05) in T2D-AT-EC. However, we found that the inhibition of tube formation by BiPS was greater in T2D-AT-EC treated with [ND-GROα] compared to [T2D- GROα] (p < 0.01) (Figure 4).

## Discussion

Adipose tissue is perhaps the most plastic tissue in the body, adjusting its mass in response to changing patterns of substrate availability, environmental stresses, neuro-hormonal stimuli, or pathological conditions. A key feature of adipose tissue remodelling is the development of an appropriate vascular network. Indeed, evidence suggests that the development of the vascular supply is a rate-limiting step in AT expansion [19–21]. Failure to adequately accomplish this goal can lead to several consequences, including reduced exposure to hormones and substrates, hypoxia, increased tissue inflammation, and, ultimately, insulin resistance [3,4]. The importance of adequate AT blood flow (ATBF) is emphasized by the observation that AT CD is reduced in obesity and insulin resistance states [3,5] and impairments in ATBF may be apparent before insulin resistance [4,22]. In adult AT, expansion of the vascular network occurs through the multistep process of angiogenesis [23]. Major determinants of the efficiency of AT angiogenesis are the presence of pro- and anti-angiogenic factors in the AT microenvironment [4,8,9,24] and the performance of resident endothelial cells [6,7].

In addition to the crucial mechanical and metabolic functions of skeletal muscle, it has become apparent over the past 20 years that muscle can also serve as a secretory tissue, releasing protein factors from myotubes termed myokines that can act in autocrine, paracrine, and endocrine fashions to regulate multiple processes (reviewed in [12,25]). A number of these myokines, such as VEGF, IL8, IL15 and GROα, are known to exert pro-angiogenic activities. Multiple investigators have shown that the secretion of selected myokines can be regulated by the presence of T2D (reviewed in [12,25]). We reported that myotubes from T2D subjects, compared to ND myotubes, release elevated amounts of multiple myokines, including IL8, IL15 and GRO, while VEGF secretion was similar between groups [11,14]. Indeed, IL8 and GRO are two of the most abundantly secreted myokines [11,14]. Thus, it is possible that these specific myokines could, by acting in a paracrine manner, contribute to the regulation of angiogenesis in skeletal muscle. Yet, while T2D myotubes release elevated levels of these pro-angiogenic factors, skeletal muscle tissue in T2D is characterized by a reduced CD [26], like that observed in AT [21]. Previously we tested possible explanations for this paradox, using the HUVEC line as a surrogate for muscle endothelial cells. Exposure of HUVEC to media conditioned by ND (ND-CM) and T2D (T2D-CM) myotubes, resulted in stimulation of capillary tube formation. However, the response to T2D-CM was considerably less than that to ND-CM [17]. As IL8, IL15 and GRO were all present at higher levels in T2D-CM, we examined the effects of these factors, applied individually or in combination at concentrations matching the average values in ND- and T2D-CM. Multiple lines of evidence revealed that it was IL8 that was responsible for the biphasic dose-dependent effect on capillary tube formation by HUVEC [16]. Thus, it is possible that the lower CD seen in T2D SkM could be due, at least in part, to the higher levels of IL8 present in the microenvironment of diabetic muscle.

Given the high levels of IL8 and GROα secreted by myotubes and the mass of skeletal muscle, it is possible that delivery of these myokines into the circulation could contribute in an endocrine manner to regulation of angiogenesis in AT as well. This postulate was tested in the current work. Important differences from our previous work include the fact that the present studies include an additional variable, the identity of the response system, AT from ND and T2D subjects, and not a homogenous system such as HUVEC. Unlike the situation with HUVEC, there was not a dose-dependent angiogenic response by AT, in this instance capillary outgrowth, to the IL8+ IL15+ GROα combination. Both HUVEC and AT responded similarly to ND- and T2D equivalent levels of IL15 alone [16]. The most striking differences between HUVEC and skeletal muscle explants and AT involved responses to IL8 and GROα. While T2D related levels of IL8 reduced angiogenesis in HUVEC and skeletal muscle explants, it had very modest transient effects in AT. Distinctly in AT it was the T2D-related higher level of GROα that blunted angiogenesis. Furthermore, it is only T2D-AT and T2D-AT-EC that displayed a differential sensitivity to GROα.

Mixed effects of GROα on cell migration and angiogenesis have been observed in multiple systems. In HUVEC, GROα primarily stimulates cytoskeletal reorganization [27] and cell migration and tube formation [28] in a dose-dependent manner, with a tendency for responses to be blunted at higher concentrations. However, in mast cells [29] and human airway smooth muscle cells [30] GROα inhibits cell migration. Little is known about possible roles of GROα in T2D [31]. Circulating levels are reported to be elevated in obese [32] and T2D subjects [33] by some, but not all [34], investigators. GROα is synthesized and secreted equally by the adipocyte and stromal-vascular fractions of omental adipose tissue (OAT) [35]. Such secretion is higher in tissue from obese individuals [35], yet the absolute amount of GROα secreted from OAT appears to much less than that produced by myotubes [14,15,36,37]. Considering the relative masses of OAT and SkM in humans, it is possible that myotube-derived GROα contributes a major portion of the GROα present in the circulation and within tissues.

GROα and IL8 share several characteristics, belonging to the same structural class of chemokines (CXC with a Glu-Leu-Arg sequence in the N-terminus) and interacting with the same receptors [25,27]. This similarity gives rise to the question of why in T2D-AT and T2D-AT-EC there is a dose-dependency for angiogenesis in response to GROα, but not IL8, while in HUVEC the opposite is true [16]. Cell type specificity in the preference between GROα and IL8 is not without precedent, as in human lung microvascular endothelial cells, IL8 is considerably more potent than GROα in stimulating actin reorganization [27]. Further support for a ‘functional distinction’ [38] between GROα and IL8 is provided by studies in rabbit knee joints, where IL8 is more potent for neutrophil recruitment, while it is GROα that induces TNFα expression [38].

Both our previous work [16] and the current findings show the varying effects of the microenvironments created by ND and T2D myokines on capillary tube formation. What the current work adds is information about the tissue selectivity of responses to specific myokines. Since AT is a highly heterogeneous system, we isolated endothelial cells from AT to determine if they were responsible for the response to GROα specific to T2D. We found that differential sensitivity to GROα is an intrinsic property of T2D-EC. Altered behaviour of T2D-EC would agree with multiple in vitro studies showing that endothelial progenitor cells (EPC) from individuals with T2D generated EC with reduced proliferation, adhesion, and capacity to form tubes, thereby inhibiting their ability to re-vascularize damaged tissue [7]. Additionally, such behaviour is observed in EC isolated from db/db mice [39] and can also be generated in vitro by culture of EC under hyperglycaemic conditions [40]. Furthermore, Gealekman and colleagues have reported that the angiogenic capacity and CD of SAT is decreased with morbid obesity [3]. The ND and T2D subjects studied here display a similar extent of adiposity, reducing the impact of that factor.

Angiogenesis is a multistep process that requires EC activation, migration, proliferation, and tube formation [41]. To allow EC migration, degradation of the extracellular matrix (ECM) surrounding the EC is required, mediated by matrix metalloproteinases (MMPs) released from EC. The reduced release of MMP-2 and −9 from T2D-AT and T2D-AT-EC after exposure to [T2D-GROα] (Figure 3) could contribute at an early step to the impaired tube formation observed. This behaviour exclusive to T2D-EC would be consistent with reports that the presence of insulin resistance is associated with a relative reduction in MMP-9 gene expression in AT [42]. In addition, MMPs have recently been shown to act on several substrates in addition to ECM proteins [41]. One of these is GROα, which is inactivated by MMP-9. Thus, reduced MMP-9 release by T2D-AT-EC in response to T2D-related levels of GROα would lead to reduced GROα cleavage, elevating GROα levels even further.

Our results reinforce the concept that T2D can influence ATBF in several ways. One is the well-documented resistance of EC to insulin-induced vasodilation [43]. Another is elevated secretion of GROα, to levels that could impair angiogenesis in AT. This would result in a reduced vascular reserve leading to reduced delivery of insulin, substrates, and oxygen to adipocytes, leading to metabolic dysfunction and hypoxia, the later triggering recruitment of inflammatory cells [4,21,43]. This could induce adipocyte apoptosis, with further release of inflammatory mediators. The importance of appropriate ATBF to metabolic health is highlighted by numerous reports demonstrating associations between reduced ATBF or CD and insulin resistance, as well as the development of T2D [4,21,43].

Several aspects of this study need to be considered about their impact on the physiologic relevance of our findings. One is a potential limitation of studies with primary cells or cell lines, including the current work, such that they may not be fully reflective of the behaviour of that specific cell type in the context of the intact tissue. Our findings with explant AT in culture confirm the results with EC in a more physiologic system and are consistent with the reduced ATCD and ATBF seen in vivo in insulin-resistant humans and rodents. Second, is that our observations about myokine effects on AT angiogenesis are limited to the factors studied, GROα, IL8 and IL15, and do not include the primary pro-angiogenic agent, VEGF. We chose to focus on myokines whose release differed between ND and T2D myotubes; unlike with VEGF [11,14]. Of note, VEGF release has been reported to be higher from AT of subjects with T2D [44], suggesting that the microenvironment of T2D AT could include elevated levels of VEGF. Potential T2D-related differential sensitivity to the angiogenic actions of VEGF represents an exciting area for further study. Circulating (serum) GROα levels in healthy humans have been reported to range over 50–90 pg/mL [32], while levels in individuals with T2D display levels ~50% higher [33]. However, this observation is not universal [34]. Also, there is limited information about absolute levels in skeletal muscle or adipose tissue, especially in humans, although it has been reported that isolated macrophages and pre-adipocytes differentiated from human OAT secrete GRO and that such secretion is elevated several-fold in cells from obese individuals or with exposure to inflammatory agents [45], thereby supporting the supposition that T2D AT may be exposed in vivo to elevated levels of GROα.

In summary, the current report reveals an intrinsic property of T2D-AT-EC whereby elevated levels of GROα reduce the angiogenic response and, ultimately, CD in AT. Our findings suggest that both the overproduction of GROα and an altered response of T2D-AT-EC to this factor can contribute to the metabolic and vascular dysfunction of AT in T2D. Meanwhile, both ND and T2D-AT respond similarly to IL8 and IL15.

## Patients/methods/materials

### Patients

AT samples were obtained from 14 ND (ND-AT) and 15 T2D (T2D-AT) subjects. Subjects were classified as ND based on values of either HbA1c<5.7 or 2 hr OGTT [glucose]<140 mg/dL within 2 months of biopsy. They also had to be negative for a family history (1^st^ degree relative) of T2D. Subjects with T2D were identified based on an existing clinical diagnosis. Anti-diabetic medications used included: metformin, glipizide, glargine and liraglutide, as well as combinations of the same. Medication use was stable for at least 3 months and maintained up to the time of biopsy. General inclusion criteria for all subjects were: weight stable (±2 kg) over the last 2 months and no medication changes over the last 3 months. Potential subjects were excluded if they were taking medications known to influence carbohydrate metabolism, including anti-depressants, and the presence of other conditions known to influence carbohydrate metabolism. Female subjects who were not post-menopausal were biopsied during the early follicular phase of their cycles. Characteristics of the subjects are summarized in Table 1. The Committees on Human Investigation of the University of California, San Diego, and VA San Diego Healthcare System approved the experimental protocol. Informed written consent was obtained from all subjects after explanation of the protocol.

**Table 1.** Subject Characteristics

Group	N (F/M)	Age (yr)	BMI (kg/m^2^)	Fasting glucose (mM)	Fasting insulin (pM)	HOMA-IR
ND	14 (4/10)	48 ± 4	33.6 ± 1.6	5.28 ± 0.16	56 ± 14	1.2 ± 0.3
T2D	15 (5/10)	47 ± 3	37.0 ± 2.4	7.94 ± 0.86†	172 ± 68	2.6 ± 0.6*

*p < 0.05 vs ND, †p < 0.01 vs ND

### Adipose tissue biopsy

Subcutaneous adipose tissue (SAT) samples (n = 20) were obtained from the lateral abdominal wall region (peri-umbilical) after an overnight (10–12 hr) fast [46]. Briefly, lidocaine (1%) was infiltrated in a square field fashion and the biopsy taken, using a 5-mm side-cutting needle, from the centre of the field. For nine subjects, SAT was obtained during elective gastric bypass surgery. In that instance, SAT samples were collected from the periumbilical region intraoperatively during dissection of the usually placed trocar sites for the laparoscopic operation. All subjects were weight stable before tissue collection.

### Ex vivo angiogenesis assay of adipose tissue

The assay was performed employing established protocols [3,5,19]. Briefly, freshly harvested human AT was cut into ~1 mm^3^ pieces, matched in size for each individual subject, which were then embedded individually in wells of a 24-well plate, in growth-factor-depleted Matrigel (280 µL/well) (BD Bioscience) and cultured in 1:1 EGM-2-MV (Lonza) and α-MEM (Life-Technologies/Gibco,). Explants were treated with rIL8 (#208-IL-010/CF), rGROα (#275–GR–010), and rIL15 (#247-ILB-005) (all recombinant proteins are human low-endotoxin from R&D Systems). Media and treatments were replaced every other day. Routinely, 3 independent explants per subject for each condition were embedded. Each explant was analysed for capillary outgrowth length by light microscopy and images captured with a 4X objective. The length of capillary branches at the periphery of the growth area was quantified by 3 separate blinded observers (for each explant), using the NIH Image-J program [3,5,19]. In control studies, the basal growth with 1:1 EGM-2-MV or α -MEM without supplements was minor. It has been demonstrated that >90% of sprouting cells from the AT explants express endothelial cell markers, and thus represent angiogenic growth [3,5,19].

### Endothelial cell isolation and identification

Endothelial cells were obtained through two different protocols. With AT collected from abdominal surgeries, the stromal-vascular fraction (SVF) was isolated by collagenase digestion, filtration and centrifugation as described previously [47,48]. CD31 positive cells were isolated with CD31-magnetic microbeads using MACS technology (Miltenyl Biotec, #130-091-935) according to the manufacturer’s instructions. With this procedure 96.2% of the cells were CD31 + . Alternatively, freshly harvested human adipose tissue was cut into ~1 mm^3^ pieces, which were embedded individually in wells of a 24-well plate, in growth-factor-depleted Matrigel (280 µL/well) and cultured in DMEM with D-Valine replacing L-Valine and supplemented with 15% FBS (GE Healthcare) [19,49,50]. After 5 days, the explant was removed, and the cells allowed to propagate for an additional 3–5 days. Cells were released from Matrigel with dispase and transferred to EGM-2-MV media. Endothelial cells were authenticated for each individual preparation using antibodies against human Von Willebrand Factor (Dako, catalogue # A0082) and PECAM-1 (CD31) (Millipore, catalogue #04-1074A). AT-EC were not tested for mycoplasma contamination. The samples were stained with Vectastain Universal Elite Kit (Vector Laboratories, #PK-6200) and DAB Substrate Kit (Vector Laboratories, #SK-4100) according to the manufacturer’s instructions.

### Culture of primary isolated endothelial cell and angiogenesis assay

Isolated EC were grown according to standard protocols in EGM-2-MV [51]. Measurements of capillary-like tube formation by the isolated cells were achieved using an in vitro assay of endothelial cell tube formation [51]. Matrigel was plated onto 96-well plates (50 µL/well) and incubated at 37°C for 60 minutes. Cells were cultured in EGM-2-MV in the absence or presence of rGROα, and then seeded onto Matrigel at 10,000 cells/well for 8 h. Cells were analysed for capillary number by light microscopy and phase contrast images captured on a Nikon TS100 microscope with a 4X objective. The readout for this assay is the formation of capillary-like tubes. A tube is defined as a closed network unit or an intact loop. Results were obtained by counting the number of tubes in a field. The number of capillaries was quantified by 3–4 separate, blinded observers, using NIH Image-J. In control studies, the extent of capillary formation with serum free EGM-2-MV was negligible. Each experiment was repeated at least 3 times in quadruplicate. In some experiments BiPS (Millipore) a specific inhibitor of MMP-2/MMP-9 [52], was added together with other treatments.

### Media collection and immunobloting

Tissue explants and isolated EC were cultured and treated with or without rGROα in the presence of EGM-2-MV under the same conditions as described above for the respective angiogenesis assays. Conditioned media was collected after 4d and 8 h, respectively, centrifuged for 10 min at 800 × g to remove cell debris and stored at −80°C before analysis. Proteins were resolved on 10% SDS-PAGE under reducing conditions, transferred to nitrocellulose membranes, blocked with Odyssey block (LI-COR Biosciences) and incubated with antibodies against MMP2 or MMP9 (Cell Signalling, catalogue #4022 and #3852, respectively). Secondary IRDye antibodies were obtained from LI-COR (catalogue #926-68,020 and #926-68,073). Detection and quantification of band intensity was performed using Odyssey Infrared Imaging System and Image Studio analysis software (version 3.1.4).

### Data analysis

Statistical analysis was performed using GraphPad Prism 5.0 (GraphPad). Between group comparisons were evaluated by independent group t test if data was normally distributed and with a Mann-Whitney test for non-normally distributed data. Variances were similar between groups. Within group comparisons (treatment effects) were evaluated by paired t test. For results that were not normally distributed, data was log-transformed for statistical analysis and then back-transformed and reported in original units as mean ± SD. Variation within each group of data was unknown at the initiation of studies. Statistical significance was accepted as p < 0.05. The number of individual determinations for each measurement is indicated in the legends. The number of individual determinations for each measurement was established after initial measurements revealed the variation for that specific measurement and set to detect a difference of 1.5 SD.

## Supplementary Material

Supplemental Material

## Data Availability

The data that support the findings of this study are available by request of the corresponding author.
