# Supplementary material for: Adipose tissue from subjects with type 2 diabetes exhibits impaired capillary formation in response to GROα: involvement of MMPs-2 and -9
Source: Adipocyte. 2022 May 12;11(1):276–86. doi: 10.1080/21623945.2022.2070949 (PMC9116416; doi:10.1080/21623945.2022.2070949)
Supplement: Supplemental Material [file KADI_A_2070949_SM3120.zip › supplementary/KADI_2021_0103_Supplemental_Info.docx]

Supplemental Material

Figure legends

**Figure SM 1.** Effects of combined or individual exogenous myokines on capillary length in ND- and T2D-AT. Quantification of capillary length of adipose tissue explants from ND-AT (A, C, E) or T2D-AT (B, D, F) after incubation for 5 or 6 days with a mixture of rIL8 + rGROα + rIL15 (A, B), rIL15 alone (C, D), and rIL8 alone (E, F), in concentrations equivalent to those present in CM from ND (solid bars) or T2D (open bars) myotubes. Average + SD, ND-AT, n=14 and T2D-AT n=15. Shown is the average capillary length/500μm^2^ for 3 fields of at least 3 different explants for each subject.

**Figure SM 2.** Characterization of endothelial cells isolated from subcutaneous adipose tissue. Representative images of endothelial cells isolated from adipose tissue stained with control IgG (a & c) or antibodies against Von Willebrand Factor (b) or PECAM-1 (CD31) (d). Magnification: a & b- 40X, c & d- 100X. e. Representative image of tube formation on Matrigel by endothelial cells isolated from subcutaneous adipose tissue (40X).
