# Supplementary figures and images for: Adipose tissue from subjects with type 2 diabetes exhibits impaired capillary formation in response to GROα: involvement of MMPs-2 and -9
Source: Adipocyte. 2022 May 12;11(1):276–86. doi: 10.1080/21623945.2022.2070949 (PMC9116416; doi:10.1080/21623945.2022.2070949)

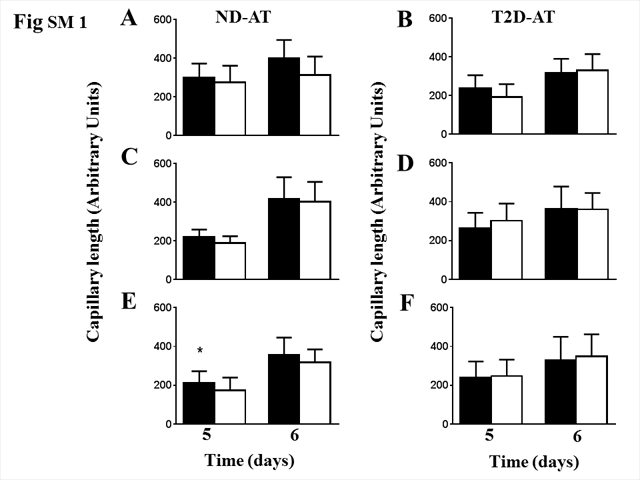

Supplement: Supplemental Material [file KADI_A_2070949_SM3120.zip › supplementary/KADI_2021_0103_Sup_Fig_1.TIFF]

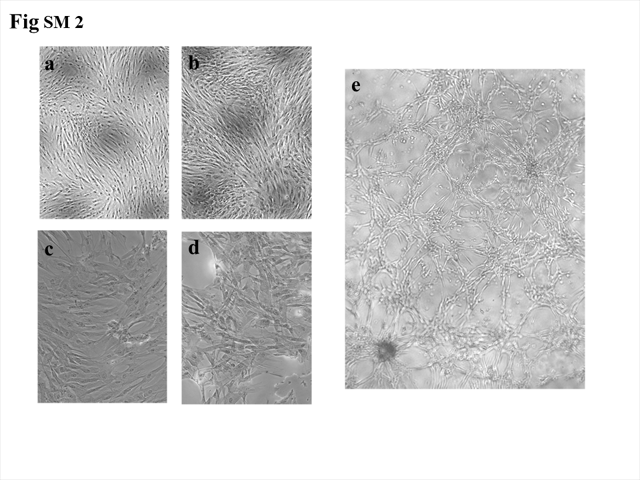

Supplement: Supplemental Material [file KADI_A_2070949_SM3120.zip › supplementary/KADI_2021_0103_Sup_Fig_2.TIFF]
